# Motor signatures of emotional reactivity in frontotemporal dementia

**DOI:** 10.1038/s41598-018-19528-2

**Published:** 2018-01-18

**Authors:** Charles R. Marshall, Chris J. D. Hardy, Lucy L. Russell, Camilla N. Clark, Rebecca L. Bond, Katrina M. Dick, Emilie V. Brotherhood, Cath J. Mummery, Jonathan M. Schott, Jonathan D. Rohrer, James M. Kilner, Jason D. Warren

**Affiliations:** 1 Dementia Research Centre, Department of Neurodegenerative Disease, Institute of Neurology, University College London, Queen Square, London, WC1N 3BG UK; 2 Sobell Department of Motor Neuroscience and Movement Disorders, Institute of Neurology, University College London, Queen Square, London WC1N 3BG UK

## Abstract

Automatic motor mimicry is essential to the normal processing of perceived emotion, and disrupted automatic imitation might underpin socio-emotional deficits in neurodegenerative diseases, particularly the frontotemporal dementias. However, the pathophysiology of emotional reactivity in these diseases has not been elucidated. We studied facial electromyographic responses during emotion identification on viewing videos of dynamic facial expressions in 37 patients representing canonical frontotemporal dementia syndromes versus 21 healthy older individuals. Neuroanatomical associations of emotional expression identification accuracy and facial muscle reactivity were assessed using voxel-based morphometry. Controls showed characteristic profiles of automatic imitation, and this response predicted correct emotion identification. Automatic imitation was reduced in the behavioural and right temporal variant groups, while the normal coupling between imitation and correct identification was lost in the right temporal and semantic variant groups. Grey matter correlates of emotion identification and imitation were delineated within a distributed network including primary visual and motor, prefrontal, insular, anterior temporal and temporo-occipital junctional areas, with common involvement of supplementary motor cortex across syndromes. Impaired emotional mimesis may be a core mechanism of disordered emotional signal understanding and reactivity in frontotemporal dementia, with implications for the development of novel physiological biomarkers of socio-emotional dysfunction in these diseases.

## Introduction

Motor mimicry supports the decoding of perceived emotions by the healthy brain^1,2^. Viewing emotional facial expressions rapidly and involuntarily engages the facial muscles of neurologically normal observers^3,4^. Emotional mimesis may have evolved as a specialized ‘exaptation’ of action observation, and by promoting emotional contagion and affective valuation may have facilitated the development of advanced human social behaviour and theory of mind^2,5,6^. In line with this interpretation, motor recoding of observed emotion correlates with empathy and emotion identification ability^7^ and predicts authenticity judgments on facial expressions^8^; while conversely, facial paralysis induced by botulinum toxin attenuates emotional reactivity^9^. The linkage between emotion observation, recognition and mimesis is precise: viewing of universal facial emotional expressions^10^ produces signature profiles of electromyographic (EMG) activity in the facial muscles conveying each expression^3,11^. This phenomenon is mediated by distributed, cortico-subcortical brain regions that may together instantiate a hierarchically organised neural substrate for inferring the intentions and subjective states of others^12–15^: primary visual representations of emotions would comprise the lowest level of the hierarchy, ascending through sensorimotor representations of emotional movement kinematics, prediction of movement goals and affective states, and encoding of intentions, including affective mentalising.

On clinical, pathophysiological and neuroanatomical grounds, altered motor recoding might be anticipated to underlie impaired emotional and social signal processing in the frontotemporal dementias (FTD). This diverse group of neurodegenerative diseases manifests as three canonical clinico-anatomical syndromes^16^; behavioural variant (bvFTD), semantic variant primary progressive aphasia (svPPA) and nonfluent variant primary progressive aphasia (nfvPPA). These broad syndromic groupings encompass various sub-syndromes: in particular, within the heterogeneous bvFTD syndrome at least two major variants can be defined, based on the relative selectivity of right temporal lobe atrophy^17,18^. Deficits in emotion recognition, empathy and social understanding and behaviour are defining features of bvFTD but integral to all FTD syndromes^19–24^ and collectively engender substantial distress and care burden^25^. Impaired facial emotion recognition in bvFTD, svPPA and nfvPPA has been linked to atrophy of an overlapping network of cerebral regions including orbitofrontal cortex, posterior insula and antero-medial temporal lobe^23,26^, implicated in evaluation of facial emotional expressions and integration with bodily signals^27–29^. Moreover, various abnormalities of physiological reactivity have been documented in FTD, including changes in resting skin conductance and heart rate variability in bvFTD and altered homeostatic and affective autonomic responses in bvFTD, svPPA and nfvPPA^30–36^. Patients with bvFTD have been noted to have reduced facial expressivity^37^ and indeed, deficient volitional imitation of emotional faces^38^. However, whereas impaired facial EMG reactivity to facial expressions has been linked to emotion processing deficits in Parkinson’s disease^39,40^, Huntington’s disease^41^ and schizophrenia^42^, the motor physiology of emotional reactivity has not been addressed in the FTD spectrum.

In this study, we investigated facial motor responses to viewing facial emotional expressions in a cohort of patients representing all major phenotypes of FTD (bvFTD, svPPA and nfvPPA) relative to healthy older individuals. In addition to the canonical syndromic FTD variants, we identified a subset of patients presenting with behavioural decline and selective right temporal lobe atrophy (right temporal variant, rtvFTD): this entity has been proposed previously to account for much of the heterogeneity of the broader bvFTD syndromic spectrum and is associated with particularly severe disturbances of facial empathy^18,38,43,44^. We compared facial EMG response profiles with emotion identification accuracy on a stimulus set comprising video recordings of dynamic, natural facial expressions: such expressions are more faithful exemplars of the emotions actually encountered in daily life and are anticipated to engage mechanisms of motor imitation more potently than the static images conventionally used in neuropsychological studies^45,46^. Neuroanatomical associations of facial expression identification and EMG reactivity in the patient cohort were assessed using voxel-based morphometry (VBM). Based on previous clinical and physiological evidence^3,4,30,31,33,34,36,37,43,47^, we hypothesised that healthy older individuals would show rapid and characteristic patterns of facial muscle responses to perceived emotional expressions coupled with efficient emotion identification. In contrast, we hypothesised that all FTD syndromes would be associated with impaired emotion identification but would exhibit separable profiles of facial muscle reactivity. In particular, we predicted that bvFTD and rtvFTD would be associated with reduced EMG responses while svPPA would be associated with aberrant coupling of muscle reactivity to emotion identification and nfvPPA with a more selective, emotion-specific reactivity profile. Based on previous neuroimaging studies both in the healthy brain and in FTD^14,23,26,45,48–50^, we further hypothesised that facial emotion identification and EMG reactivity would have partly overlapping neuroanatomical correlates within the extensive cortical circuitry previously implicated in the decoding of visual emotional signals, supplementary motor and insular cortices mediating the integration of somatic representations and antero-medial temporal and prefrontal circuitry involved in the evaluation of emotion. Within these distributed networks (given the known neuroanatomical heterogeneity of the target syndromes) we predicted a differential emphasis of grey matter correlates, with more marked involvement of inferior frontal, anterior cingulate and insular cortices in bvFTD and nfvPPA and more extensive, lateralised temporal lobe involvement in svPPA and rtvFTD^16–18^.

## Materials and Methods

### Participants

Thirty-seven consecutive patients fulfilling consensus criteria for a syndrome of FTD^51,52^ (19 with bvFTD, nine with svPPA, nine with nfvPPA) and 21 healthy older individuals with no history of neurological or psychiatric illness participated. General characteristics of the participant groups are summarised in Table 1. No participant had a history of facial palsy or clinically significant visual loss after appropriate correction. There was clinical evidence of orofacial apraxia in seven patients in the nfvPPA group, but none in any of the other participant groups. General neuropsychological assessment (see Table 1) and brain MRI corroborated the syndromic diagnosis in all patients; no participant had radiological evidence of significant cerebrovascular damage. Based on visual inspection of individual brain MR images, six patients with a behavioural syndrome and relatively selective right temporal lobe atrophy were re-categorised as a rtvFTD subgroup (throughout this paper, we use ‘bvFTD’ to refer to those patients with a behavioural presentation not re-classified as rtvFTD). Between group differences in demographic and neuropsychological variables were analysed using ANOVAs with post hoc T-tests when main effects were found, except for categorical variables, for which a chi-squared test was used.

**Table 1:** Demographic, clinical and neuropsychological characteristics of participant groups.

Characteristic	Controls	bvFTD	rtvFTD	svPPA	nfvPPA
Demographic/clinical					
No. (male:female)	9:12	10:3	6:0^a^	7:2	4:5
Age (years)	69.1 (5.3)	66.2 (6.3)	63.8 (6.4)	66.1 (6.5)	69.6 (6.5)
Handedness (R:L)	20:1	12:1	6:0	8:1	7:2
Education (years)	15.7 (3.5)	13.2^c^ (2.5)	18.0 (3.1)	14.9 (2.8)	15.0 (2.1)
MMSE (/30)	29.6 (0.6)	24.5^a^ (4.6)	25.3^a^ (4.3)	21.8^a^ (6.9)	23.7^a^ (6.0)
Symptom duration (years)	N/A	7.7 (6.0)	6.5 (3.5)	5.6 (3.0)	4.7 (2.2)
Neuropsychological					
*General intellect*					
WASI Verbal IQ	125 (6.7)	89^a^ (21.9)	87^a^ (22.2)	77^a^ (19.7)	80^a^ (17.3)
WASI Performance IQ	125 (10.2)	104^a^ (20.3)	107 (24.6)	108 (23.5)	99^a^ (21.5)
*Episodic* *memory*					
RMT words (/50)	49.0 (1.4)	37.4^a^ (7.9)	37.2^a^ (9.3)	30.0^a,c^ (6.3)	41.4^a^ (9.5)
RMT faces (/50)	44.7 (3.5)	33.5^a^ (6.9)	34.8^a^ (7.9)	32.8^a^ (6.9)	39.5 (6.6)
Camden PAL (/24)	20.4 (3.3)	10.8^a^ (8.1)	12.5^a^ (6.2)	2.2^a,b,c,e^ (3.7)	16.3 (7.8)
*Executive* *skills*					
WASI Block Design (/71)	44.8 (10.5)	32.5 (16.7)	37.2 (22.1)	39.1 (21.7)	25.1^a^ (19.7)
WASI Matrices (/32)	26.6 (3.9)	19.3^a^ (9.4)	19.0^a^ (9.8)	19.8^a^ (10.6)	17.4^a^ (9.0)
WMS-R DS forward (max)	7.1 (1.1)	6.6 (1.2)	6.8 (1.2)	6.7 (1.2)	4.8^a,b,c,d^ (0.8)
WMS-R DS reverse (max)	5.6 (1.2)	4.0^a^ (1.5)	4.7 (1.4)	5.3 (1.8)	3.0^a, d^ (0.7)
D-KEFS Stroop:					
color (s)	33.4 (7.2)	48.0 (20.5)	48.8 (21.4)	53.2^a^ (28.2)	87.0^a,b,c,d^ (6.7)
word (s)	23.9 (5.6)	32.5 (19.0)	38.7 (26.1)	36.0 (24.0)	85.4^a,b,c,d^ (10.3)
interference (s)	57.6 (16.7)	99.6^a^ (47.5)	98.3 (45.1)	90.1 (56.1)	165^a,b,c,d^ (30.8)
Fluency:					
letter (F total)	18.1 (5.6)	7.8^a^ (4.6)	9.0^a^ (4.7)	8.9^a^ (7.1)	3.5^a^ (1.7)
category (animals total)	24.4 (6.0)	13.8^a^ (7.5)	10.3^a^ (2.3)	5.7^a,b^ (5.1)	8.8^a^ (3.5)
Trails A (s)	33.7 (7.3)	56.5 (32.3)	59.8 (32.9)	49.7 (20.1)	81.7^a^ (48.4)
Trails B (s)	67.3 (21.5)	171.7^a^ (88.2)	186.7^a^ (100.4)	134.9 (101.7)	211.1^a^ (94.6)
*Language* *skills*					
WASI Vocabulary	72.3 (3.2)	42.4^a^ (21.5)	47.0^a^ (19.1)	33.6^a^ (22.0)	31.7^a^ (13.9)
BPVS	148.6 (1.1)	120.8 (38.7)	141.8 (7.2)	85.8^a,b,c,e^ (53.8)	142.6 (10.1)
GNT	26.1^a^ (2.7)	12.2^a^ (10.2)	12.5 (10.1)	1.6^a,b,c,e^ (4.7)	15.5^a^ (6.6)
*Other* *skills*					
GDA (/24)	15.8 (5.3)	7.8^a^ (6.6)	7.5^a^ (6.3)	11.9 (8.6)	5.4^a^ (1.9)
VOSP (/20)	19.0 (1.5)	15.9^a^ (3.4)	16.7 (2.3)	15.8 (4.5)	15.3^a^ (4.7)

Mean (standard deviation) scores are shown unless otherwise indicated; maximum scores are shown after tests (in parentheses). ^a^significantly different from healthy controls, ^b^significantly different from bvFTD, ^c^significantly different from rtvFTD, ^d^significantly different from svPPA, ^e^significantly different from nfvPPA (all p < 0.05). BPVS, British Picture Vocabulary Scale (Dunn LM *et al*., 1982); bvFTD, patient group with behavioural variant frontotemporal dementia (excluding right temporal cases); Category fluency totals for animal category and letter fluency for the letter F in one minute (Gladsjo *et al*., 1999); Controls, healthy control group; D-KEFS, Delis Kaplan Executive System (Delis *et al*., 2001); DS, digit span; GDA, Graded Difficulty Arithmetic (Jackson M and Warrington, 1986); GNT, Graded Naming Test (McKenna and Warrington, 1983); MMSE, Mini-Mental State Examination score (Folstein *et al*., 1975); N/A, not assessed; NART, National Adult Reading Test (Nelson, 1982); nfvPPA, patient group with nonfluent variant primary progressive aphasia; PAL, Paired Associate Learning test (Warrington, 1996); RMT, Recognition Memory Test (Warrington, 1984); rtvFTD, patient subgroup with right temporal variant frontotemporal dementia; svPPA, patient group with semantic variant primary progressive aphasia; Synonyms, Single Word Comprehension: A Concrete and Abstract Word Synonyms Test (Warrington *et al*., 1998); Trails-making task based on maximum time achievable 2.5 minutes on task A, 5 minutes on task B (Lezak *et al*., 2004); VOSP, Visual Object and Spatial Perception Battery – Object Decision test (Warrington and James, 1991); WAIS-R, Wechsler Adult Intelligence Scale‐-Revised (Wechsler, 1981); WASI, Wechsler Abbreviated Scale of Intelligence (Wechsler, 1997); WMS, Wechsler Memory Scale (Wechsler, 1987).

This study was approved by the University College London institutional ethics committee and all methods were performed in accordance with the relevant guidelines and regulations. All participants gave informed consent in accordance with the Declaration of Helsinki.

### Facial expression stimuli

Videos of emotional facial expressions were obtained from the Face and Gesture Recognition Research Network (FG-NET) database^53^. This database comprises silent recordings of healthy young adults viewing emotional scenarios: the scenarios were designed to elicit spontaneous, canonical facial expressions, but were presented without any instruction to pose or inhibit particular expressions (further details in Supplementary Material). In order to sample the spectrum of facial expressions, for each of the canonical emotions of anger, fear, happiness, surprise and disgust^10^ we selected 10 videos (50 stimuli in total; see Table S1) that clearly conveyed the relevant expression (the canonical emotion of sadness was omitted because its more diffuse time course sets it apart from other emotional expressions). Each video stimulus lasted between four and eight seconds (mean 4.9 seconds), commencing as a neutral facial expression and evolving into an emotional expression (further information in Supplementary Material). The video frame in which an emotional expression first began to develop unambiguously from the neutral baseline (previously determined by independent normal raters and provided with the FG-NET database) was used to align data traces across trials.

Stimuli were presented in randomised order via the monitor of a notebook computer running the Cogent toolbox of Matlab R2012b. The participant’s task on each trial was to identify from among the five alternatives (verbally or by pointing to the appropriate written name) which emotion was displayed; participant responses were recorded for offline analysis. Participants were first familiarised with the stimuli and task to ensure they understood and were able to comply with the protocol. During the test, no feedback was given and no time limits were imposed on responses. Emotion identification scores were compared among groups using ANOVAs, with Bonferroni-corrected post hoc T-tests when main effects were found.

### EMG acquisition and analysis

While participants viewed the video stimuli, facial EMG was recorded continuously from left corrugator supercilii, levator labii and zygomaticus major muscles with bipolar surface electrodes, according to published guidelines for the use of EMG in research^54^. These facial muscles were selected as the key drivers of the canonical expressions represented by the video stimuli^3,11^. Expressions of anger and fear engage corrugator supercilii (which knits the brow) and inhibit zygomaticus major (which raises the corner of the mouth); expressions of happiness and surprise are associated with the reverse muscle activity profile, while disgust engages both corrugator supercilii and levator labii (which curls the top lip). EMG data were sampled at 2048Hz with a 0.16–100Hz band-pass filter and the EMG signal was rectified, high-pass filtered to correct for baseline shifts and smoothed with a 100 data point sliding filter using MATLAB R2012b; trials with signal amplitude >3 standard deviations from the mean (attributable to large artifacts, e.g., blinks) were removed prior to analysis. For each trial, the mean change in EMG activity from baseline (mean activity during a 500 ms period prior to trial onset) was analysed for each muscle in 500 ms epochs, starting 1s before the onset of expression change in the video stimuli; the EMG response for each muscle was calculated as the area under the curve of EMG signal change from baseline.

We first assessed the presence of automatic imitation (any EMG change from baseline) and emotion-specific muscle activation (any interaction of muscle EMG response with emotion) for the healthy control group, using a repeated measures ANOVA (mean EMG activity for five emotions in eight 500 ms time bins for the three muscles). To determine if there was an overall effect of participant group on the degree of emotion-specific muscle activation, EMG responses were compared across all participants using a restricted maximum likelihood mixed effects model incorporating interactions between emotion, muscle and participant group, with participant identity as a level variable and time bin as a covariate of no interest. After assessing the overall effect of participant group in the omnibus test, we proceeded to establish the basis for any group differences by examining particular emotion-specific muscle contrasts. Emotion-specific EMG response profiles were quantified for each trial by combining individual muscle responses pairwise as follows: for anger and fear, (corrugator response minus zygomaticus response); for happiness and surprise, (zygomaticus response minus corrugator response); for disgust, (corrugator response plus levator response). These pairwise muscle contrasts have been shown to improve reliability and internal consistency of facial EMG analysis^55^. Muscle contrast EMG reactivity for each trial was then analysed as a dependent variable in an ANOVA incorporating participant group and emotion as fixed factors. Significant main effects in the ANOVA were explored with post hoc T-tests, using Bonferroni correction for multiple comparisons.

To test the hypothesis that emotional imitation supports identification, we assessed any relationship between overall EMG reactivity and emotion identification score using Spearman’s rank correlation across the participant cohort. In addition, we compared EMG responses on trials with correct versus incorrect emotion identification and assessed any interaction with participant group membership using an ANOVA.

To generate an overall measure of reactivity for each participant for use in the voxel based morphometry analysis, EMG reactivity was averaged over all trials for that participant and then normalised as the square root of the absolute value of the change in muscle activity from baseline (subzero values corresponding to muscle activity changes in the reverse direction to that expected were restored).

For both emotion recognition and EMG reactivity, we assessed correlations with neuropsychological instruments indexing general nonverbal intellectual ability (nonverbal executive performance on the WASI Matrices task) and semantic knowledge (performance on the British Picture Vocabulary Scale), to examine the extent to which the experimental parameters of interest were influenced by disease severity and background semantic deficits.

For all tests, the criterion for statistical significance was thresholded at p < 0.05.

### Brain image acquisition and analysis

Each patient had a sagittal 3-D magnetization-prepared rapid-gradient-echo T1-weighted volumetric brain MR sequence (echo time/repetition time/inversion time 2.9/2200/900 msec, dimensions 256 256 208, voxel size 1.1 1.1 1.1 mm), acquired on a Siemens Trio 3T MRI scanner using a 32-channel phased-array head-coil. Pre-processing of brain images was performed using the New Segment^56^ and DARTEL^57^ toolboxes of SPM8 (www.fil.ion.ucl.ac.uk/spm), following an optimised protocol^58^. Normalisation, segmentation and modulation of grey and white matter images were performed using default parameter settings and grey matter images were smoothed using a 6 mm full width-at-half-maximum Gaussian kernel. A study-specific template mean brain image was created by warping all bias-corrected native space brain images to the final DARTEL template and calculating the average of the warped brain images. Total intracranial volume was calculated for each patient by summing grey matter, white matter and cerebrospinal fluid volumes after segmentation of tissue classes.

Processed brain MR images were entered into a VBM analysis of the patient cohort. Separate regression models were used to assess associations of regional grey matter volume (indexed as voxel intensity) with mean overall emotion identification score and EMG reactivity, for each syndromic group. Age, total intracranial volume and WASI Matrices score (a measure of nonverbal executive function and index of disease severity) were incorporated as covariates of no interest in all models. Statistical parametric maps of regional grey matter associations were assessed at threshold p < 0.05 after family-wise error (FWE) correction for multiple voxel-wise comparisons within pre-specified regional volumes of interest. For the emotion identification contrast, these regions were informed by previous studies of emotion processing in FTD and in the healthy brain, comprising insula, anteromedial temporal lobe (including amygdala, fusiform gyrus and temporal pole), inferior frontal cortex, anterior cingulate and supplementary motor cortices^23,26,48^. For the EMG reactivity contrast, regions of interest were based on previous functional imaging studies of facial mimicry and dynamic facial stimuli^14,45,49,50^, comprising visual (V1, MT/V5, parahippocampal and fusiform gyri) and primary and supplementary motor cortices.

## Results

### General characteristics of participant groups

General clinical characteristics of the participant groups are presented in Table 1. There was a significant gender difference between participant groups (chi^2^_4_ = 10.31, p = 0.036), but no significant age difference. The patient groups did not differ in mean symptom duration or level of overall cognitive impairment (as indexed using WASI Matrices score; ANOVAs and post hoc T-tests all p > 0.4).

### Emotion identification

Group data for facial emotion identification are summarised in Table 2.

**Table 2:** Summary of emotion identification and EMG reactivity findings for participant groups

Response parameter	Controls	bvFTD	rtvFTD	svPPA	nfvPPA
Emotion identification					
Anger	4.6 (2.2)	1.8 (1.4)^a^	2.5 (1.6)	1.1 (0.9)^a^	3.4 (1.7)
Disgust	8.1 (1.0)	5.3 (3.3)^a^	3.5 (3.9)^a^	3.8 (3.3)^a^	5.4 (3.3)
Fear	5.4 (2.1)	2.6 (2.0)^a^	2.0 (1.7)^a^	3.9 (2.0)	4.4 (2.4)
Happiness	9.2 (0.8)	8.0 (3.2)	8.3 (1.9)	7.0 (3.2)	7.8 (1.6)
Surprise	8.4 (1.0)	4.9 (2.8)^a^	3.7 (2.8)^a^	4.1 (3.2)^a^	5.8 (3.0)
*Overall* (*/50*)	35.7 (4.6)	22.7 (9.5)^a^	20.0 (9.7)^a^	20.2 (7.9)^a^	26.9 (9.3)^a^
Facial EMG reactivity					
Anger	1.3 (3.3)	0.5 (1.5)	0.2 (1.0)	1.2 (5.1)	0.3 (4.0)
Disgust	2.6 (8.9)	−0.9 (9.0)^a^	0.5 (1.7)	1.4 (6.2)	0.9 (3.7)
Fear	0.7 (2.9)	0.3 (1.3)	−0.1 (1.9)	0.8 (4.4)	−0.9 (3.5)^a,b,c^
Happiness	1.3 (2.3)	0.5 (1.3)^d^	0.2 (1.6)^d^	1.8 (8.2)	2.3 (4.9)
Surprise	1.0 (2.5)	0.01 (3.1)^c,d^	0.3 (1.8)	1.7 (5.3)	1.7 (3.8)
*Overall*	1.4 (4.7)	0.09 (4.4)^a,c,d^	0.2 (1.6)^a,c^	1.4 (6.0)	0.9 (4.2)

**Figure 1:**
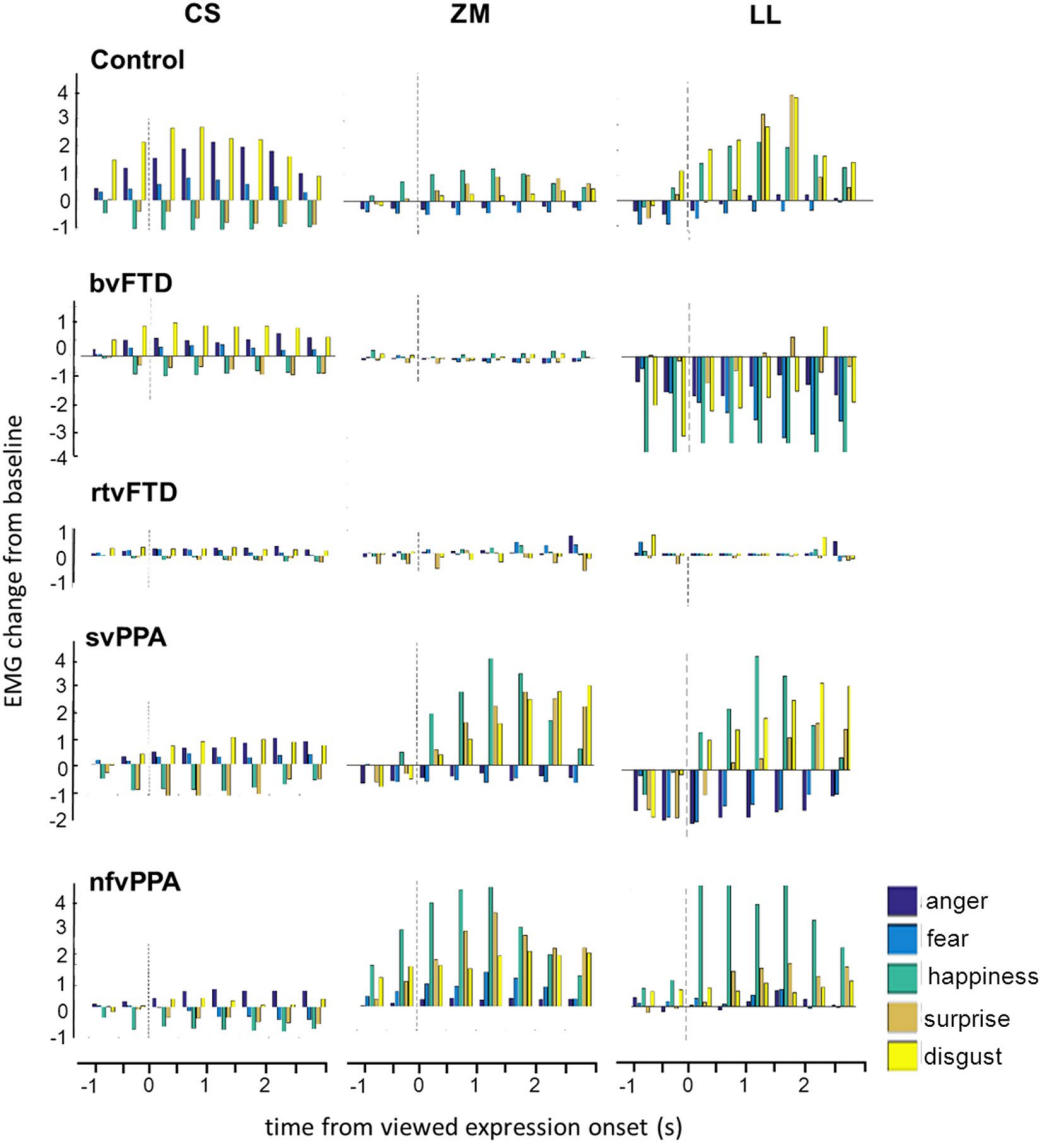
Patterns of EMG reactivity for each muscle in each participant group. For each participant group, the plots show the time course of average EMG reactivity (in microvolts) for key facial muscles while participants watched videos of emotional facial expressions. EMG reactivity, here indexed in arbitrary units as mean EMG change from baseline, is shown on the y-axis (after rectifying, high-pass filtering and removing artefacts as described in Methods). Onset of the viewed facial expression (as determined in a prior independent analysis of the video stimuli) is at time 0 (dotted line) in each panel. In healthy controls, corrugator supercilii (CS) was activated during viewing of anger, fear and disgust, but inhibited during viewing of happiness and surprise; zygomaticus major (ZM) was activated during viewing of happiness and surprise, but inhibited during viewing of anger and fear; and levator labii (LL) was inhibited during viewing of anger and fear, and maximally activated during viewing of disgust. Note that in healthy controls muscle responses consistently preceded the unambiguous onset of viewed emotional expressions. bvFTD, patient group with behavioural variant frontotemporal dementia (excluding right temporal cases); Control, healthy control group; nfvPPA, patient group with nonfluent variant primary progressive aphasia; rtvFTD, patient subgroup with right temporal variant frontotemporal dementia; svPPA, patient group with semantic variant primary progressive aphasia.

Mean (standard deviation) scores on the emotion identification task and mean facial EMG reactivity (as defined in Fig. 1) to viewed emotional expressions are shown for each emotion, in each participant group. ^a^significantly less than healthy controls, ^b^significantly less than bvFTD, ^c^significantly less than svPPA, ^d^significantly less than nfvPPA (all p_bonf_ < 0.05). bvFTD, patient group with behavioural variant frontotemporal dementia (excluding right temporal cases); Controls, healthy control group; nfvPPA, patient group with nonfluent variant primary progressive aphasia; rtvFTD, patient subgroup with right temporal variant frontotemporal dementia; svPPA, patient group with semantic variant primary progressive aphasia.

Overall accuracy of facial emotion identification showed a main effect of participant group (F_4_ = 10.89, p < 0.001), and was reduced in all syndromic groups relative to controls (all p_bonf_ < 0.012) (Table 2). There was no significant relationship between emotion identification accuracy and age but a significant effect of gender (p = 0.04), with higher identification scores overall in female participants. The main effect of participant group persisted after covarying for gender (F_4_ = 13.852, p < 0.001). Emotion identification accuracy in the patient cohort correlated with standard measures of nonverbal executive function (WASI Matrices score, an index of disease severity; rho = 0.547, p < 0.001) and semantic competence (British Picture Vocabulary Scale; rho = 0.676, p < 0.001).

### Facial EMG reactivity

**Figure 2:**
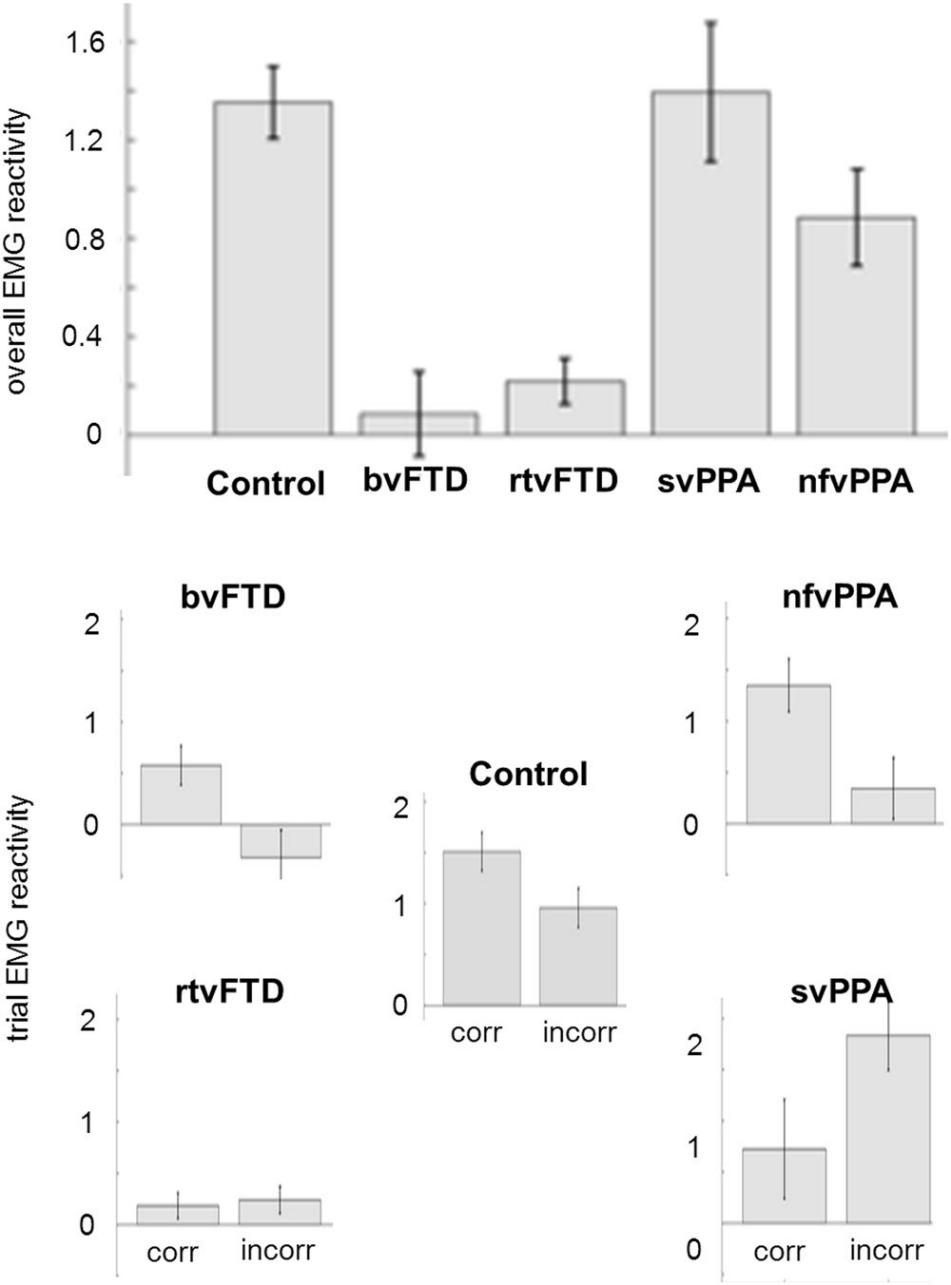
EMG reactivity in each participant group, and the relationship with identification accuracy. For each participant group, the histograms show mean overall facial muscle EMG reactivity (top) and EMG reactivity separately (below) for those trials on which viewed emotional expressions were identified correctly (corr) versus incorrectly (incorr); error bars indicate standard error of the mean (see also Table 2). bvFTD, patient group with behavioural variant frontotemporal dementia; Control, healthy control group; nfvPPA, patient group with nonfluent variant primary progressive aphasia; rtvFTD, patient subgroup with right temporal variant frontotemporal dementia; svPPA, patient group with semantic variant primary progressive aphasia.

Mean time courses of EMG responses for each facial muscle and emotion are shown for all participant groups in Fig. 1. Group data for EMG reactivity are summarised in Table 2 and Fig. 2.

Healthy older participants showed the anticipated profiles of facial muscle activity in response to viewing facial expressions (Fig. 1): corrugator supercilii was activated by anger, fear and disgust, and inhibited by happiness and surprise; zygomaticus major was activated by happiness and surprise, and inhibited by anger and fear; and levator labii activity was maximal for disgust. Due to the proximity of levator labii and zygomaticus major, and the limited spatial specificity of surface electrodes^54^, there was substantial electrical leakage between these two muscles. However, zygomaticus major was maximally activated by happiness and surprise, and levator labii by disgust; moreover, these muscles were not combined in any of the pairwise muscle contrasts.

EMG reactivity to viewed facial expressions was modulated in an emotion- and muscle-specific manner in healthy controls (F_(2.20,43.94)_ = 5.03, p = 0.009) and the participant cohort as a whole (chi^2^_(8)_ =  80.05, p < 0.001). There was further evidence that this interaction between emotion and muscle reactivity varied between participant groups (interaction of group, emotion and muscle: (chi^2^_(32)_ = 143.91, p < 0.001). After the generation of a muscle contrast reactivity measure for each trial, ANOVA revealed significant main effects of participant group (F_(4)_ = 10.84, p < 0.001), emotion (F_(4)_ = 3.40, p = 0.009) and the interaction of group and emotion (F_(16)_ = 2.79, p < 0.001; Table 2). In post hoc T-tests comparing participant groups (with Bonferroni correction), overall EMG reactivity across the five emotions was significantly reduced in the bvFTD group relative to the healthy control group (p_bonf_  < 0.001), the svPPA group (p_bonf_ < 0.001) and the nfvPPA group (p_bonf_ = 0.042); and significantly reduced in the rtvFTD group relative to the healthy control group (p_bonf_ = 0.001) and the svPPA group (p_bonf_ = 0.005).

There was no significant relationship between EMG reactivity and age (p = 0.1), gender (p = 0.42), or WASI Matrices score (used here as a measure of disease severity; p = 0.63) in the patient cohort, nor with a standard measure of semantic knowledge (British Picture Vocabulary Scale; p = 0.5).

### Relationship between emotion identification and facial EMG reactivity

Across the participant cohort, overall EMG reactivity was significantly correlated with emotion identification accuracy (rho = 0.331, p = 0.011) and mean trial EMG reactivity was significantly higher for trials on which the emotion was correctly identified (n = 1586) than on error trials (n = 1314; p = 0.002). This differential effect of correct versus incorrect trials showed a significant interaction with participant group (F_(4)_ = 4.18, p = 0.002; see Fig. 2). Among healthy controls, there was a strong trend towards greater reactivity predicting correct identification (p = 0.087). Comparing trial types within patient groups, EMG reactivity was significantly higher on correct identification trials than error trials in the bvFTD group (p = 0.009) and the nfvPPA group (p = 0.01) but not the rtvFTD group (p = 0.76) or the svPPA group (p = 0.06, here signifying a trend towards greater EMG reactivity on incorrect trials).

### Neuroanatomical associations

**Figure 3:**
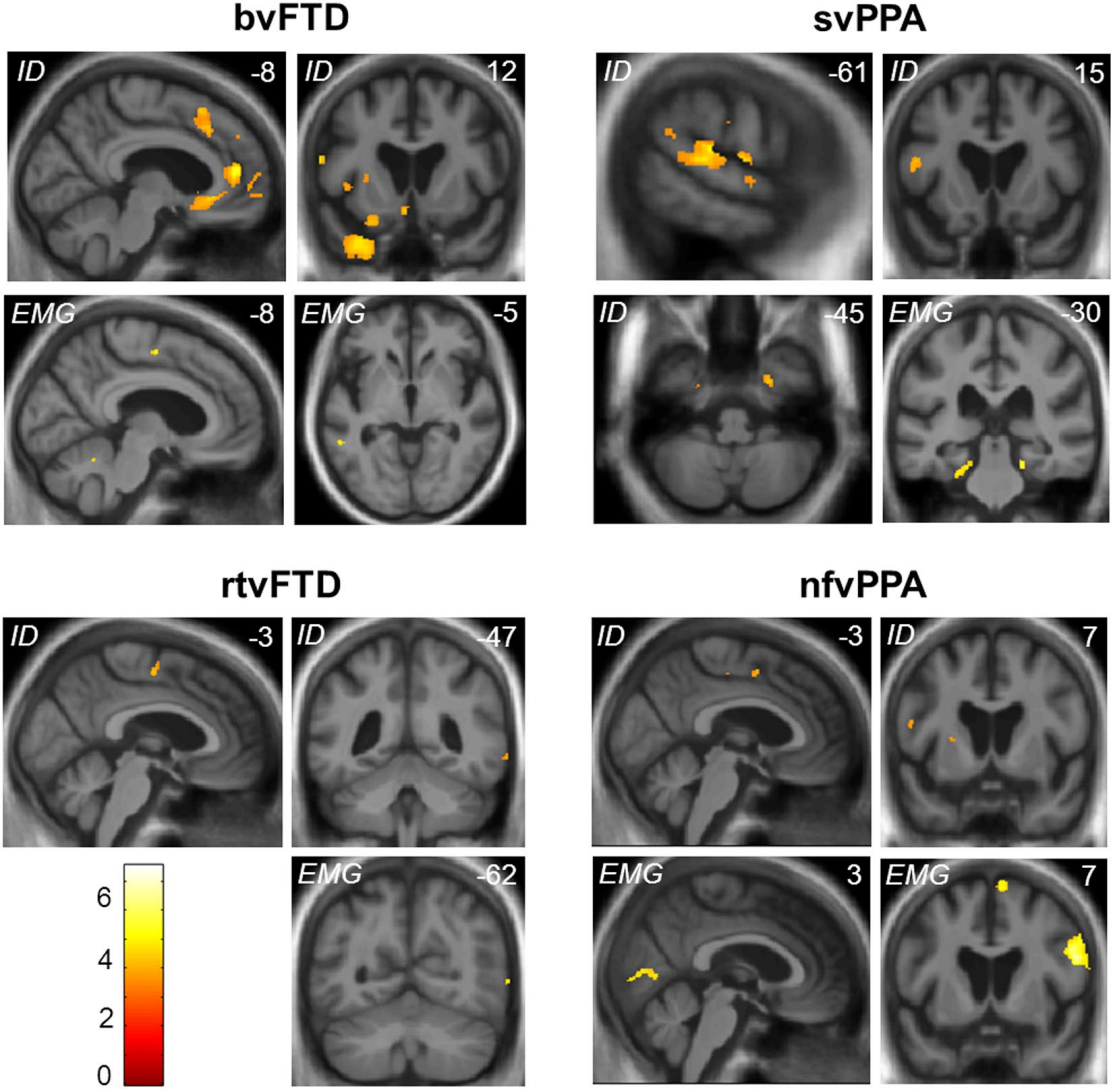
Neuroanatomical correlates of emotion identification and EMG reactivity for each syndromic group. Statistical parametric maps (SPMs) show regional grey matter volume positively associated with overall emotion identification accuracy and facial EMG reactivity during viewing of emotional facial expressions, based on voxel-based morphometry of patients’ brain MR images (see also Table 3); T-scores are coded on the colour bar. SPMs are overlaid on sections of the normalised study-specific T1-weighted mean brain MR image; the MNI coordinate (mm) of the plane of each section is indicated (coronal and axial sections show the left hemisphere on the left). Panels code syndromic profiles of emotion identification (*ID*) or EMG reactivity (*EMG*). Note that the correlates of emotion identification and EMG reactivity in different syndromes overlapped in particular brain regions, including supplementary motor cortex and temporo-occipital junction (see Table 3). SPMs are thresholded for display purposes at p < 0.001 uncorrected over the whole brain, however local maxima of areas shown were each significant at p < 0.05 after family-wise error correction for multiple voxel-wise comparisons within pre-specified anatomical regions of interest (see Table 3). bvFTD, patient group with behavioural variant FTD; nfvPPA, patient group with nonfluent variant primary progressive aphasia; rtvFTD, patient subgroup with right temporal variant frontotemporal dementia; svPPA, patient group with semantic variant primary progressive aphasia.

Significant grey matter associations of emotion identification and EMG reactivity for the patient cohort are summarised in Table 3 (all thresholded at p_FWE_ < 0.05 within pre-specified anatomical regions of interest); statistical parametric maps are presented in Fig. 3.

**Table 3:** Neuroanatomical correlates of emotion identification and reactivity in patient groups.

Group	Region	Side	Cluster	Peak (mm)			T score	P_FWE_
			(voxels)	x	y	z		
*Emotion identification*								
bvFTD	Anterior cingulate	L	196	−8	44	12	5.59	0.003
	Anterior insula	L	123	−30	27	0	4.07	0.047
	Supplementary motor area	L	5	−10	4	50	3.81	0.044
	Opercular IFG	L	32	−57	12	18	5.14	0.003
	Anteromedial temporal:							
	Temporal pole	L	2133	−32	8	−38	5.11	0.010
	Amygdala			−24	2	−38	4.94	0.015
	Fusiform gyrus			−30	−9	−38	4.82	0.019
rtvFTD	Supplementary motor area	L	34	−3	−10	57	4.15	0.022
	Temporo-occipital junction	R	18	66	−50	−8	4.06	0.038
svPPA	STG/STS	L	536	−58	−30	14	7.21	0.005
	Supplementary motor area	L	19	−4	−2	50	4.23	0.019
	Opercular IFG	L	25	−57	12	18	5.05	0.003
	Anterior cingulate	L	24	−2	44	3	4.11	0.042
	Fusiform gyrus	R	44	22	−4	−44	4.43	0.042
nfvPPA	Supplementary motor area	L	37	−4	−2	50	4.14	0.023
	Opercular IFG	L	9	−52	8	18	3.96	0.033
*Facial EMG reactivity*								
bvFTD	Supplementary motor area	L	12	−8	−9	56	3.99	0.030
	Temporo-occipital junction	L	25	−54	−45	−4	4.29	0.064
rtvFTD	Temporo-occipital junction	R	8	62	−62	2	3.96	0.046
svPPA	Parahippocampal gyrus	L	59	−20	−28	−24	4.25	0.028
	Parahippocampal gyrus	R	72	18	−33	−18	5.25	0.003
nfvPPA	Primary visual cortex	R	291	12	−80	3	5.92	0.001
	Primary motor cortex	R	521	56	8	27	5.43	0.007
	Supplementary motor area	R	18	8	8	68	4.42	0.012

The table presents regional grey matter correlates of mean overall emotion identification score and facial EMG reactivity (as defined in Fig. 1) during viewing of facial expressions in the four patient groups, based on voxel-based morphometry. Coordinates of local maxima are in standard MNI space. P values are all significant after family-wise error (FWE) correction for multiple voxel-wise comparisons within pre-specified anatomical regions of interest (see text). bvFTD, patient group with behavioural variant frontotemporal dementia (excluding right temporal cases); IFG, inferior frontal gyrus; nfvPPA, patient group with nonfluent variant primary progressive aphasia; rtvFTD, patient subgroup with right temporal variant frontotemporal dementia; STG/S, superior temporal gyrus/sulcus; svPPA, patient group with semantic variant primary progressive aphasia.

Accuracy identifying dynamic emotional expressions was correlated with regional grey matter volume in left supplementary motor cortex in all syndromic groups. Additional regional grey matter correlates of emotion identification were delineated for particular syndromic groups. The bvFTD, svPPA and nfvPPA groups showed syndromic grey matter correlates within a bi-hemispheric (predominantly left-lateralised) frontotemporal network including opercular inferior frontal gyrus, anterior cingulate, anterior insula and antero-inferior temporal lobe; while the svPPA group showed a further correlate in left posterior superior temporal cortex and the rtvFTD group showed a correlate in right temporo-occipital junctional cortex in the vicinity of MT/V5 complex^59^.

Across the patient cohort, overall mean EMG reactivity was correlated with regional grey matter in an overlapping but more posteriorly directed and right-lateralised network, with variable emphasis in particular syndromic groups. The bvFTD and nfvPPA groups showed grey matter correlates of EMG reactivity in supplementary and primary motor cortices, while all syndromic groups showed grey matter associations in cortical areas implicated in the analysis of visual signals, comprising primary visual cortex in the nfvPPA group; temporo-occipital. junction (MT/V5 complex) in the bvFTD and rtvFTD groups; and parahippocampal gyrus in the svPPA group.

## Discussion

Here we have demonstrated facial motor signatures of emotional reactivity in the FTD spectrum. As anticipated, healthy older individuals showed characteristic profiles of facial muscle engagement by observed facial emotions; moreover, facial muscle reactivity predicted correct trial-by-trial identification of facial emotions. These findings provide further evidence that (in the healthy brain) facial mimesis is an automatic, involuntary mechanism supporting stimulus decoding and evaluation, rather than simply an accompaniment of conscious emotion recognition. In contrast, overall facial muscle reactivity and the normal coupling of muscle reactivity to facial emotion identification were altered differentially in the patient groups representing major FTD syndromes. As predicted, identification of facial expressions was impaired across the patient cohort: however, whereas the bvFTD group showed globally reduced facial muscle reactivity to observed emotional expressions, the svPPA group had preserved overall muscle reactivity but loss of the linkage between muscle response and correct expression identification. Among those patients with syndromes dominated by behavioural decline, the profile of facial muscle reactivity stratified cases with rtvFTD from other cases of bvFTD: the subgroup with rtvFTD had a particularly severe phenotype, exhibiting both globally reduced facial reactivity and also aberrant coupling of muscle reactivity to facial expression identification.

Considered collectively, the motor signatures of emotional reactivity identified in our patient cohort amplify previous clinical, neuropsychological and physiological evidence in particular FTD syndromes. The generalised impairment of emotional mimesis in our bvFTD and rtvFTD groups is consistent with the clinical impression of facial impassivity^37,60^, impaired intentional imitation^38^ and blunting of autonomic responsiveness^30,31,33,35,36^ in these patients. Abnormal coupling of facial mimesis to facial expression identification in our svPPA group is in line with the disordered autonomic signalling of affective valuation previously documented in this syndrome^33,35^, and suggests a method of dissociating emotional reactivity from the declarative, semantic categorisation of emotions. The present findings suggest that aberrant motor recoding of perceived expressions may constitute a core physiological mechanism for impaired emotion processing in FTD.

This mimetic mechanism may be particularly pertinent to the dynamically shifting and subtle emotions of everyday interpersonal encounters. Our own emotional expressions are normally subject to continual modulation by the expressed emotions of others, including tracking of transient ‘micro-expressions’^61^; this modulation occurs over short timescales (a few hundred milliseconds) and contributes importantly to the regulation of social interactions, prosociality and empathy^28,62–64^. If facial mimesis plays a key role in tuning such responses, loss of this modulatory mechanism (most notably in bvFTD and rtvFTD) might underpin not only impaired socio-emotional awareness in FTD but also the ‘poker-faced’ sense of unease these patients commonly provoke in others^37^.

The neuroanatomical correlates we have identified speak to the coherent nature of dynamic emotion mimesis and identification. In line with previous evidence^38^, these processes mapped onto a distributed cerebral network within which FTD syndromes showed separable profiles of grey matter atrophy. Involvement of supplementary motor cortex was a feature across syndromes and associated both with emotion identification and motor reactivity, though joint correlation was observed in the bvFTD and nfvPPA groups but not the rtvFTD and svPPA groups (see Table 3). Supplementary motor cortex is a candidate hub for the computation of sensorimotor representations unfolding over time, an integral function of the mirror neuron system: this region generates both facial sensory-evoked potentials and complex facial movements^65^ and it is activated during facial imitation and empathy^66^ as well as by dynamic auditory emotional signals^48^. Furthermore, transcranial magnetic stimulation of the supplementary motor region disrupts facial emotion recognition^67^. The uncoupling of motor reactivity from emotion identification in the rtvFTD and svPPA groups may reflect disconnection of this key hub from linked mechanisms for affective semantic appraisal^12^, perhaps accounting for lack of an EMG reactivity correlate in supplementary motor cortex in these syndromic groups. Two further cortical hubs correlating both with emotion identification and mimesis were delineated in our patient cohort. In the svPPA and rtvFTD groups, a joint correlate was identified in the temporo-occipital junction zone, overlapping posterior superior temporal sulcus and MT/V5 visual motion cortices^59,68^: this region has been implicated in the imitation and decoding of dynamic facial expressions^15,49,69,70^, integration of dynamic social percepts, action observation and theory of mind^71,72^. In the svPPA group, infero-medial temporal cortex was linked both to emotion identification and mimesis: this region has previously been shown to respond to dynamic facial stimuli^45^.

Additional grey matter associations of facial expression identification accuracy were delineated in cingulo-insular, antero-medial temporal and inferior frontal areas previously implicated both in the detection and evaluation of salient affective stimuli and in canonical FTD syndromes^15,20,21,23,26,73,74^. Additional grey matter associations of facial motor reactivity were identified (for the nfvPPA group) in primary visual and motor cortices: enhanced responses to emotional facial expressions have previously been demonstrated in visual cortex^75^, while motoric responses to social stimuli have been located in precentral gyrus^14^. However, it is noteworthy that certain grey matter associations emerging from this analysis - in particular, the ‘hub regions’ of supplementary motor cortex and temporo-occipital junction and (in the nfvPPA group) primary visual and motor cortices - lie beyond the brain regions canonically targeted in particular FTD syndromes or indeed, in previous studies of emotion processing in FTD^21^. It is likely that the dynamic expression stimuli employed here allowed a more complete picture of the cerebral mechanisms engaged in processing naturalistic emotions. Moreover, involvement of brain regions remote from zones of maximal atrophy may reflect distributed functional network effects (for example, visual cortical activity has been shown to be modulated by amygdala^75^) in conjunction with disease-related network connectivity changes, which are known to extend beyond the atrophy maps that conventionally define particular FTD syndromes^76^. Taken together, the present neuroanatomical findings are compatible with the previously proposed, hierarchical organisation of embodied representations supporting emotional decoding and empathy^13,48,77,78^: whereas early visual and motor areas may support automatic imitation via low-level visual and kinematic representations, higher levels of the processing hierarchy engage the human ‘mirror’ system and substrates for semantic, evaluative and mentalising processes that drive explicit emotion identification.

From a clinical perspective, this work suggests a pathophysiological framework for deconstructing the complex social and emotional symptoms that characterise FTD syndromes. Such symptoms are difficult to measure using conventional neuropsychological tests, and may only be elicited by naturalistic social interactions: dynamic motor physiological surrogates might index both the affective dysfunction of patients’ daily lives and the underlying disintegration of culprit neural networks^38^. These physiological metrics might facilitate early disease detection and tracking over a wider spectrum of severity than is currently possible and enable socio-emotional assessment in challenging clinical settings (such as aphasia), especially since our results suggest that (in contrast to explicit emotion recognition) automatic motor reactivity may be relatively insensitive to semantic deficits. Our findings further suggest that such metrics are not simply ciphers of reduced cognitive capacity but may help stratify broad disease groupings (such as the heterogeneous bvFTD syndrome) and at the same time, may capture mechanisms that transcend traditional syndromic boundaries. We therefore propose that the paradigm of emotional sensorimotor reactivity may yield a fresh perspective on FTD nosology and candidate novel biomarkers of FTD syndromes. Looking forward, this paradigm suggests a potential strategy for biofeedback-based retraining of emotional responsiveness, perhaps in conjunction with disease-modifying therapies^79^.

This study establishes a preliminary proof of principle but the findings require further corroboration. There are several clear limitations that suggest caution in interpreting our findings and directions for future work. We have studied a small, intensively phenotyped patient cohort: the most pressing issue will be to replicate the findings in larger clinical populations. Future studies should encompass a wider range of pathologies, in order to determine the general applicability of the paradigm and the specificity of syndromic motor profiles; it would be of interest, for example, to assess the heightened emotional contagion previously documented in Alzheimer’s disease^80^ in this context. Longitudinal cohorts including presymptomatic mutation carriers will be required in order to assess the diagnostic sensitivity of mimetic indices and their utility as biomarkers; ultimately, histopathological correlation will be necessary to establish any molecular correlates of the syndromic stratification suggested here. It will be relevant to explore the cognitive milieu of emotional motor responses in greater detail: for example, the effects of other sensory modalities (in particular, audition^48^, micro-expressions^61^, sincere versus social emotions^81^ and emotional ‘caricatures’ in FTD^82^) and the correlation of mimetic markers with measures of social cognition and daily life empathy^38^. Emotional reciprocity might be modeled using virtual reality techniques to generate model social interactions^62^. Beyond mimesis, integration of somatic and cognitive mechanisms during social emotional exchanges demands the joint processing of autonomic and neuroendocrine signals under executive control^29,83,84^: future work should assess other physiological modalities alongside EMG. Functional MRI and magnetoencephalography would amplify the present structural neuroanatomical correlates by capturing disease-related changes in underlying brain network connectivity and dynamics. Multimodal studies of this kind may set motor mimicry in the context of a comprehensive physiology of socio-emotional reactivity in neurodegenerative diseases. The ultimate goal will be to identify practical physiological markers that can be widely translated for the diagnosis and dynamic tracking of these diseases and the evaluation of new therapies.

## Electronic supplementary material

Supplementary Information
