# Supplementary material for: Motor signatures of emotional reactivity in frontotemporal dementia
Source: Sci Rep. 2018 Jan 18;8:1030. doi: 10.1038/s41598-018-19528-2 (PMC5773553; doi:10.1038/s41598-018-19528-2)
Supplement: Supplementary file 1 — Supplementary Information [file 41598_2018_19528_MOESM1_ESM.pdf]

Motor signatures of emotional reactivity in frontotemporal dementia

Charles R Marshall[1,2], Chris JD Hardy[1], Lucy L Russell[1], Camilla N Clark[1], Rebecca L Bond[1], Katrina M Dick[1], Emilie V Brotherhood[1], Cath J Mummery[1], Jonathan M Schott[1], Jonathan D Rohrer[1], James M Kilner[2*], Jason D Warren[1*]

*Joint senior authors

[1]Dementia Research Centre, Department of Neurodegenerative Disease and [2]Sobell Department of Motor Neuroscience and Movement Disorders, Institute of Neurology, University College London, Queen Square, London, UK WC1N 3BG

Corresponding author: Dr Charles Marshall, Dementia Research Centre, Institute of Neurology, Queen Square, London WC1N 3BG, UK. Email: charles.marshall@ucl.ac.uk, tel: 0203 448 3404.

Table S1. Summary of video stimuli characteristics for each emotion

Emotion	Male:Female faces	Mean duration (range) (s)
Anger	4:6	4.0 (4-4)
Disgust	5:5	6.0 (6-6)
Fear	5:5	6.2 (6-8)
Happiness	6:4	4.4 (4-6)
Surprise	6:4	4.1 (4-5)
Overall	26:24	4.9 (4-8)

For video stimuli from the FG-NET database conveying each of the universal emotional facial expressions, data for gender balance and stimulus duration are presented
